# Genotype diversity of *Mycobacterium* isolates from children in Jimma, Ethiopia

**DOI:** 10.1186/1756-0500-6-352

**Published:** 2013-09-04

**Authors:** Bereket Workalemahu, Stefan Berg, Wondewosen Tsegaye, Alemseged Abdissa, Tsinuel Girma, Markos Abebe, Abraham Aseffa

**Affiliations:** 1 Armauer Hansen Research Institute (AHRI/ALERT), Addis Ababa, Ethiopia; 2 Medical Laboratory Technology Team, Arba Minch College of Health Sciences, Arba Minch, Ethiopia; 3 Department of Laboratory Sciences and Pathology, Jimma University, Jimma, Ethiopia; 4 Bovine TB Department, Animal Health and Veterinary Laboratories Agency (AHVLA), Weybridge, UK; 5 Department of Paediatrics and Child Health, Jimma University, Jimma, Ethiopia

**Keywords:** Paediatric TB, *Mycobacterium*, Paediatric spoligotype, Gastric lavage

## Abstract

**Background:**

Paediatric tuberculosis (TB) is poorly addressed in Ethiopia and information about its magnitude and the genotype distribution of the causative *Mycobacterium tuberculosis* strains responsible for its spread are scanty.

**Methods:**

Gastric lavage or sputum samples were collected from consecutively enrolled TB suspect children visiting Jimma University Hospital in 2011 and cultured on Middlebrook 7H11 and Löwenstein-Jensen media. Acid fast bacterial (AFB) isolates were subjected to molecular typing targeting regions of difference (RDs), 16S rDNA gene and the direct repeat (DR) region using multiplex polymerase chain reaction (mPCR), gene sequencing and spoligotyping, respectively. Molecular drug susceptibility testing of *M. tuberculosis* isolates was performed by Genotype®MTBDR*plus* line probe assay (LPA) (Hain Life Sciences, Germany).

**Results:**

Gastric lavage (n = 43) or sputum (n = 58) samples were collected from 101 children and 31.7% (32/101) of the samples were positive for AFB by microscopy, culture and/or PCR. Out of 25 AFB isolates, 60% (15/25) were identified as *M. tuberculosis* by PCR, and 40% isolates (10/25) were confirmed to be non-tuberculous mycobacteria (NTM) by genus typing and 16S rDNA gene sequencing. Lineage classification assigned the *M. tuberculosis* strains into Euro-American (EUA, 66.7%; 10/15), East-African-Indian (EAI; 2/15), East-Asian (EA; 1/15) and Indio-Oceanic (IO; 1/15) lineages. Seven *M. tuberculosis* strains were new to the SpolDB4 database. All of the *M. tuberculosis* isolates were susceptible to isoniazid (INH) and rifampicin (RIF), except for one strain (of spoligotype SIT-149 or T3_ETH family) which had a mutation at the *inh*A locus which often confers resistance to INH (low level) and ethionamide.

**Conclusions:**

Analysis of the genetic population structure of paediatric *M. tuberculosis* strains suggested similarity with that of adults, indicating an on-going and active transmission of *M. tuberculosis* from adults to children in Ethiopia. There were no multidrug-resistant TB (MDR-TB) strains among the isolates.

## Background

Tuberculosis (TB) accounts for more than 10% of hospital admissions and death in children in developing countries such as Ethiopia [1-3]. The high burden of TB among children is assumed to be due to the high prevalence of TB in adults, HIV co-infection, malnutrition and other conditions related to poverty [4].

Addressing childhood tuberculosis is important not only because of the morbidity and mortality burden in children but also because it is a public health measure of recent transmission of *M. tuberculosis* and thus a sentinel indicator of the effectiveness of TB control programmes. TB infection in childhood impacts on future control strategies as a source of transmission decades later due to reactivation of latency [5]. Ethiopia is a TB high burden country (prevalence of 237/100,000 people) with low case detection rates [6]. Infection among children is likely to be similarly high since children acquire the disease mainly from adults [2,7]. The actual magnitude of paediatric TB is unknown in the country and national reports have so far provided little information on its prevalence [8].

Molecular characterization of strains has allowed for the analysis and better understanding of transmission dynamics, genetic phylogeny, strain virulence and drug resistance [9,10]. Spoligotyping is one of the widely used molecular methods for simultaneous detection and typing of *M. tuberculosis* complex bacteria and the identification of epidemiological links between patients [11], although it has less discriminatory power compared to the restriction fragment length polymorphism (RFLP) typing using insertion element IS*6110*[12].

Based on large sequence polymorphism, six main lineages have been described within the *M. tuberculosis* complex (MTBC) affecting humans: Indio-oceanic (IO) (Lineage 1), East-Asian (EA) (Lineage 2), East-African-Indian (EAI) (Lineage 3), Euro-American (EUA) (Lineage 4), West African Lineage I (Lineage 5) and West African Lineage II (Lineage 6) [13]. However, the naming and the grouping systems vary according to the molecular marker and the method of typing used. For example the SpolDB4 system assigns the MTBC organisms into 62 clades/lineages based on the genome variability at the DR locus (e.g. EAI, Beijing, CAS, Haarlem, T, X, AFRI 1 and AFRI 2) [14]. Gagneux and Small [15] have reviewed the different terminology and the genetic markers used to assign the MTBC into different phylogeographic groupings. Once the genotype data are available, different online computer programs and databases can assist in suggesting and assigning of the MTBC strains into genetic groups using a set of mathematical rules and according to the molecular marker used [16-18].

This study aimed to identify the genetic diversity of *Mycobacterium* isolates responsible for paediatric TB in Jimma University Hospital in southwest Ethiopia and compare this with already described diversity among adult Ethiopians.

## Methods

### Study population and area

Jimma University Hospital is located 355 km southwest of Addis Ababa. The hospital has 300 beds and provides curative and preventive service for 300–400 patients per day at its outpatient department [19]. The Paediatric and Child Health Department of the hospital gives inpatient and outpatient services to children (<15 years of age) and provides medical care for more than 100 children daily in the outpatient (OPD) section. Children under 15 years of age who presented to the OPD with clinically suspected tuberculosis according to the national guideline based on sign symptom complex, chest X-ray and tuberculin skin test (TST) findings were consecutively recruited into this study [20] over a period of one year in 2011.

### Ethical clearance

The study was approved by the Ethics Review Boards of Jimma University, AHRI/ALERT Ethical Review Committee (Ref No: P015/09), and the National Health Research Ethics Review Committee (NERC) of the Ethiopian Ministry of Science and Technology (Ref No: RDHE/82-92/2010). Informed consent was obtained from parents or guardians and additional assent was obtained from children older than 12 years of age.

### Sample collection and processing

Three sputum samples were collected from each child able to expectorate sputum. From younger children unable to provide sputum, gastric lavage samples were collected in the inpatient department on three consecutive mornings after an overnight fast. This was done by inserting a nasogastric tube into the stomach immediately after the patients woke up in the morning [21]. A minimum of 30 ml gastric fluid was drawn into a 50 ml syringe attached to the tube and transferred to a 50 ml sterile plastic tube. If there was no aspirate, the stomach was irrigated with 50 ml of sterile physiologic saline and aspirated back into the syringe. Within 30 minutes of sample collection, sodium bicarbonate (100 mg of sodium bicarbonate per 5 to 10 ml of aspirate) was used to neutralize the sample before storage at −20°C for a maximum of 2 months until transported on ice to the TB laboratory of the Armauer Hansen Research Institute where the samples were further stored at −20°C and processed for culture within 2 weeks of receipt.

The digestion-decontamination procedure of all collected samples was based on the method developed by Kubica *et al.*[22-25]. Briefly, a fresh solution of digestant was prepared by adding 0.5 mg of NALC powder to 100 ml of sterile NaOH and tri-sodium citrate mixture (4% NaOH and 2.9% tri-sodium citrate; Sigma). The final concentration of NaOH in the digestant was 2%. The samples were decontaminated by adding an equal volume of fresh digestant in a 50 ml sterile plastic tube for 15 minutes at room temperature, and neutralized by excess of phosphate buffered saline (PBS) pH 7.2 (Sigma, Germany). The sample was centrifuged for 15 minutes at 3200 RCF in a cooling ultracentrifuge. The supernatant was then discarded aseptically and the pellet re-suspended in 3 ml sterile PBS buffer.

### Culture

Fluka’s TB base medium was used to prepare Löwenstein-Jensen (LJ) slants according to manufacturer instructions (Fluka Chemie GmbH, Switzerland). To support the growth of *M. bovis*, one of the media contained pyruvate (Sigma, Germany). Gruft’s mycobacterial supplement (Fluka Chemie GmbH, Switzerland), which contained penicillin, nalidixic acid and ribonucleic acid, was aseptically added to all LJ media as supplement for the selective cultivation of mycobacteria according to the manufacturer’s recommendation (Fluka Chemie GmbH, Switzerland). Agar-base solid media were prepared using Difco™ Middlebrook 7H11 Agar (Difco Laboratories, USA).

Decontaminated samples were each inoculated on four LJ (supplemented with either pyruvate or glycerol) and one 7H11 agar slants. Tubes were incubated at 37°C and inspected for growth daily for the first week and weekly thereafter for eight weeks. Suspected colonies were tested for AFB by Ziehl-Neelsen staining and positive isolates were characterized by molecular techniques described below.

### Species identification

Isolates were heat killed by suspending 2–3 AFB colonies in 500 μl distilled water in a test tube and heated at 95°C submerged in an ultrasonic water bath for 1 hour. A set of six primers were used in a multiplex PCR to determine isolates for genus *Mycobacterium*, *M. tuberculosis* complex, *M. intracellulare* and *M. avium* in a single PCR assay according to Wilton and Cousins [26]. Isolates were considered as non-tuberculous mycobacteria (NTM), if they were identified as mycobacteria that did not belong to the MTBC by this multiplex PCR method. These organisms were subjected to 16S rDNA sequencing for species identification; the PCR method, amplification and detection conditions have been described previously [27]. For isolates identified as MTBC, an additional set of 3 primers targeting Region of Difference 9 (RD9) was performed to differentiate *M. tuberculosis* from other members of the MTBC.

### Spoligotyping

MTBC organisms confirmed by RD9 typing were further characterized by spoligotyping. The procedure involved heat killing isolates, PCR amplification of spacers in the DR locus and detection according to previously described protocol [10]. *M. tuberculosis* H37Rv*, M. bovis* BCG and Qiagen RNAse free water was included as positive and negative controls. The spoligotyping data was analyzed using spolTools program [28].

### GenoType®MTBDR*plus* assay

Sensitivity of the isolated *M. tuberculosis* strains to isoniazid (INH) and rifampicin (RIF) was tested with the GenoType®MTBDR*plus* line probe assay (LPA) performed according to the manufacturer’s protocol (Hain Life Science GmbH, Germany).

## Results

### Characteristics of the study participants

A total of 121 participants were included in this study and the male to female ratio was 1.3. The age of the patients ranged from one to fifteen with a mean age of 6.5 years. Forty three percent (52/121) of the participants were under 5 years of age (Table 1). All of the participants had a clinical diagnosis of TB according to the national algorithm based on a combination of clinical and laboratory findings (including cough of ≥ 2 weeks duration, contact history, tuberculin skin test positivity and chest X-ray). There was no sample for analysis from 20 participants (16.5%) due to withdrawal or laboratory rejection of gastric lavage samples containing food particles. Among the 101 cases for whom samples were available, 58 (57.4%) had provided sputum and 43 (42.6%) gastric lavage.

**Table 1:** Clinical and socio-demographic characteristics of consecutively enrolled childhood TB suspects visiting Jimma University Hospital, 2011 (n = 121)

**Risk factor**	**Category**	**No**	**%**
Age (in year)	1–5	52	43
	6–10	46	38
	11–15	23	19
Sex	Male	69	57
	Female	52	43
Contact history	Yes	67	55
	No	54	45
Fever	Yes	98	81
	No	23	19
Night sweats	Yes	103	85
	No	18	15
HIV	Reactive	15	12
	Non-reactive	106	88
Tuberculin skin test	0 mm	65	54
	1–10 mm	34	28
	>10 mm	22	18
Anthropometry	Normal	43	36
	Mild malnourished	28	23
	Moderate malnourished	29	24
	Severe malnourished	21	17

All participants in the study had cough of greater than 2 weeks of duration. The sign and symptom complex was defined as: night sweats, sweating that leads to wetting of the bed sheet; fever, body temperature of >37.5°C; close contact, living in the same household as, or in frequent contact with, a source case with sputum smear-positive pulmonary TB or clinically diagnosed TB; and anthropometry were based on weight, height/length and mid-upper arm circumference (MUAC) measurements and classification of acute malnutrition is according to Waterlow scheme (weight for height (W/H) (not malnourished, W/H >90 percent of reference median; mild malnutrition, W/H 80–90 percent of reference median; moderate acute malnutrition, W/H 70–80 percent of reference median; and severe acute malnutrition, W/H <70 percent of reference median).

### AFB microscopy and culture

**Figure 1:**
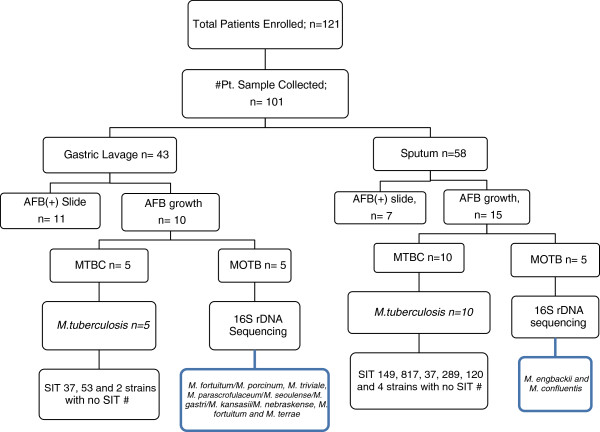
Results from bacteriological and molecular analysis of gastric lavage and sputum samples from pediatric TB suspects at Jimma University Hospital, Ethiopia, in 2011.

Two hundred and thirty samples from 101 patients (on average 2.3 samples from each child) were processed separately by culture and microscopy. Specimens from the same patient were considered together for analysis and a patient was considered positive when any one of the specimens from a patient was positive for mycobacteria. Smear microscopy was positive for AFB in 18/101 (17.8%) and mycobacterial isolates were detected in 25/101 (24.6%) patients. Gastric lavage was positive for AFB microscopy in 11 patients and culture yielded isolates in 10 cases (Figure 1).

### Molecular analysis

**Figure 2:**
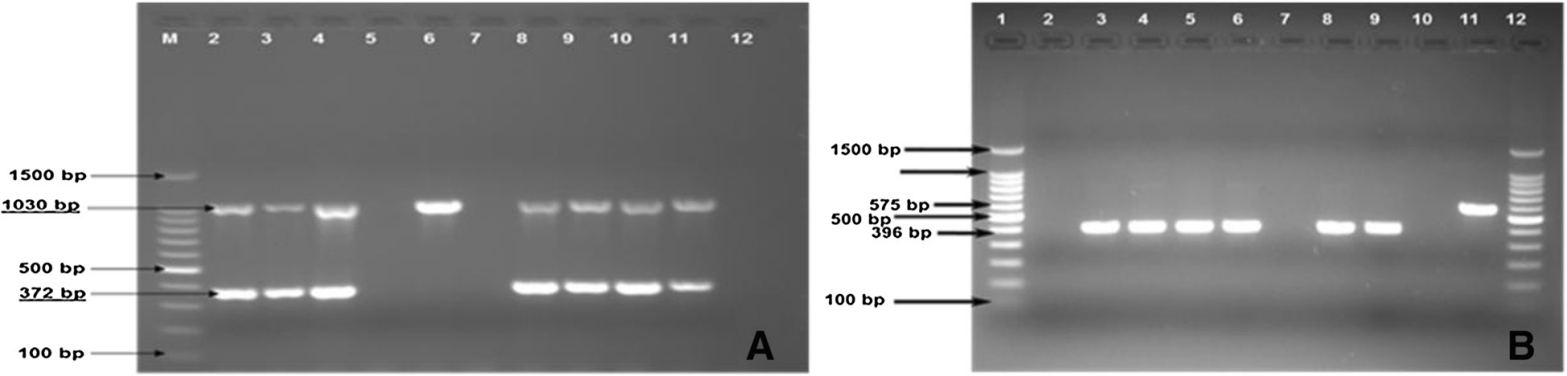
**Gel electrophoresis of multiplex Genus (A) and RD9 (B) typing for isolates of *Mycobacterium *species. A**: The multiplex Genus typing method used defines the genus *Mycobacterium*, MTBC, *M. avium* and *M. intracellulare*. Expected PCR Product for *Mycobacterium* genus, *M. intracellulare,* MTBC and *M. avium* were 1030 bp, 850 bp, 372 bp and 180 bp respectively. Lane 1, 100 bp DNA ladder; Lanes 2–9, selected isolates (Lanes 2–4, 6, 8, 9, isolates identified as of *Mycobacterium* genus; Lanes 2–4, 8, 9, isolates of MTBC; Lane 6, isolate of NTM); Lane 10–12, controls (Lane 10, *M. tuberculosis* H37Rv; Lane 11, *M. bovis* BCG; Lane 12, Negative control. **B**: RD9 typing showing expected PCR products for *M. tuberculosis* (RD9 intact, 396 bp) and for MTBC members other than *M. tuberculosis* (RD9 deleted, 575 bp). Lane 1 and 12, 100 bp DNA ladder; Lanes 2–8, selected isolates (Lanes 2 and 7, no PCR product (not of MTBC); Lane 3–6, 8, identified as *M. tuberculosis*; Lane 9–11, controls (*M. tuberculosis* H37Rv; Lane 10, negative control (H_2_O Qiagen); Lane 11, *M. bovis* AF2122/97.

**Figure 3:**
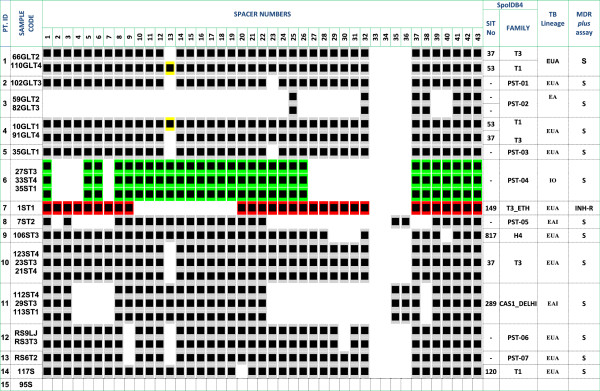
**Spoligotype pattern of *M. tuberculosis *strains isolated from children with suspected TB in Jimma, Ethiopia, 2011*. ***Spoligotype pattern of *M. tuberculosis* isolated from 14 children (one *M. tuberculosis* strain failed), also showing the corresponding SIT no, the family label (SPOLDB4) and SPOT CLUST grouping. H4, Haarlem; CAS1_DELHI, The Central Asian sub-lineage; EUA, Euro-American; EA, East-Asian; IO, Indo-Oceanic; EAI, East-African-Indian; PST, Orphan paediatric Spoligotype.

The 25 AFB organisms isolated on culture were further characterized by molecular typing. Genus typing revealed that all AFB isolates belonged to the genus *Mycobacterium*; 60% (15/25) were isolates from the MTBC and the remaining strains (10/25; 40%) were typed as NTM. 16S rDNA sequence analysis of the NTM isolates suggested *M. fortuitum/M. porcinum, M. triviale, M. parascrofulaceum*/*M. seoulense/M. gastri/M. kansasii*/*M. nebraskense, M. fortuitum, M. terrae, M. engbackii* and *M. confluentis* as the most likely species (Figure 2A). Further analysis of the MTBC strains identified all of them as *M. tuberculosis,* intact for the RD9 region (Figure 2B). Spoligotyping of these *M. tuberculosis* isolates showed that eight belonged to Shared International Type (SIT) number 37, 53, 149, 817, 289 and 120 according to the SpolDB4 database nomenclature [14], while the remaining 7 strains were new to this database. Using the Run TB-Lineage program, all (100%) of the *M. tuberculosis* strains could be accurately categorized into one of the CDC’s TB lineages: Euro-American (EUA, 66.7%; 10/15), East-African-Indian (EAI; 2/15) and East-Asian (EA; 1/15), and one strain (PST-04) probably belonging to the Indio-Oceanic (IO; 1/15) lineage with a probability of 0.94 (Figure 3). All of the *M. tuberculosis* strains tested for drug resistance were found to be susceptible to INH and RIF except for one strain (of spoligotype SIT-149 or T3_ETH family) that had mutation at the *inh*A locus which often confers resistance to INH (low level) and ethionamide.

## Discussion

The actual burden of childhood tuberculosis is unknown due to lack of routine case records and difficulty of diagnosing TB in children, both clinically and bacteriologically. However, it is estimated that in countries with high burden of TB up to 25% of the TB cases occur in children [6]. Thus, childhood tuberculosis represents a significant but still neglected clinical and public health problem [29]. Epidemiologically, investigation into paediatric TB is potentially more informative than adult TB in order to understand the status of recent transmission, evaluate effectiveness of TB control programmes or predict future trends in TB transmission [5]. Molecular tools such as spoligotyping have been standardized and widely used for species identification and depiction of the genetic diversity of MTBC organisms [10]. Using these tools, we characterized the clinical isolates of *M. tuberculosis* from paediatric patients in the Jimma area of south west Ethiopia and categorized them into previously established MTBC genetic groupings [14,30]: 10 strains as belonging to Lineage 4 (EUA), 2 as Lineage 3 (EAI), 1 as Lineage 2 (EA), and 1 as Lineage 1 (IO) according to the TB lineage program [16]. Interestingly, gastric lavage specimens were positive for AFB on smear microscopy at similar proportions as culture possibly due to limited survival of the bacilli in the acidic environment of gastric lavage.

It is well established that MTBC strains have a clonal genetic population structure and are highly restricted to geographical regions [15]. Firdessa *et al.*[31] reported the genetic population structure of the MTBC from an Ethiopian adult population that was predominantly composed of EUA (Lineage 4) and EAI (CAS, according to SpolDB4; Lineage 3). In agreement with that report, most of the strains isolated in this small series belonged to Lineage 4 (EUA) (60%) and Lineage 3 or EAI/CAS (13%). As far as can be concluded from the relatively few numbers and the single geographic area of recruitment, it seems that the population structure of our paediatric *M. tuberculosis* strains parallels that of the adult TB patients in Ethiopia. The contrary is possible as illustrated in a report on paediatric strains from Mexico, which entirely differed from the one prevailing in adult TB patients in one particular state [32]. This fact suggests active transmission of TB from adults to children in Ethiopia, since children mainly acquire the disease from adults. Unlike our dataset, genotyping of isolates from 400 culture positive children in South Africa had identified the most contagious and virulent Beijing lineage to be the predominant strain in both paediatric [33] and adult South African patients [34].

High proportions (46.7%; 7/15) of the spoligotypes from our study children were orphan patterns, i.e., patterns not previously reported to the international database of SpolDB4 (SITVIT2 database) [14]. This finding suggests that the diversity of spoligotype patterns in Ethiopia is not fully explored. A report on Mexican children had also identified that up to 34.5% of the spoligotype patterns were orphan to the SITVIT2 database [32]. Construction of an MTBC strain database dedicated to paediatric isolates would be beneficial for research on TB in children. The clinical spectrum of disease and diagnostic approaches of childhood TB are different from those of adults and the two may need to be addressed separately [35].

Two thirds of the rapidly growing mycobacterial isolates from clinical specimens could cause true to probable infections and most of them could be isolated from respiratory sites. A large proportion of the patients with respiratory NTM isolates have underlying lung injuries and co-isolation of other pathogenic microorganisms such as the *M. fortuitum* complex group (*M. fortuitum, M. porcium* and others) is common [36]. The *M. fortuitum* group has also been isolated from respiratory samples of children in our study (2/7; 28.5%). *M. kansasii* is a slow growing mycobacterium commonly found in tap water rather than in soil or salt water and consequently, *M. kansasii* related disease occurs in areas where contaminated drinking water is found [37]. Our *M. kansasii* isolate from gastric lavage could be a contaminant from the hospital tap water or an opportunistic nosocomial pathogen in children with a weakened resistance. An assessment of the mycobacteriology of the hospital environment might help clarify the source.

No multidrug-resistant TB (MDR-TB) strain was detected among the 25 paediatric TB isolates tested in this study. There was one INH-resistant strain (4%). A study on adults in the same hospital in 2010 reported a 1.5% rate of multi-drug resistance and 13.4% INH resistance [38]. Although MDR TB appears to be rare among children at the site, the presence of MDR TB in the area albeit at relatively low proportions should alert health workers to the risk of MDR TB in children complicating care in an already difficult diagnostic environment.

## Conclusions

Genetic typing of MTBC strains isolated from childhood TB patients in Jimma showed that the same lineages that dominate among adults, Lineages 4 and 3 (EUA and EAI or CAS), are prominent in children, suggesting on-going transmission of TB from adults to children in the area. A high proportion of strains were also unique to the SpolDB4 database and this calls for further studies to explore MTBC strains causing childhood TB in Ethiopia on a larger scale. To this end, constructing and populating a database dedicated to MTBC strains isolated from children is of potentially significant impact in understanding and monitoring the epidemiology of TB in children in this high burden country.

## Competing interests

All the authors declare no competing interests.

## Authors’ contributions

BW was the primary researcher, conceived the study design, participated in sample collection, performed laboratory experiment, conducted the data analysis and drafted the manuscript for publication. SB performed part of the laboratory experiment, data analysis and draft manuscript. AAs, TG, WT, MA and AAb participated in the design of the study and reviewed the initial and final drafts of the manuscript. All authors read and approved the final manuscript.
